# Immunolab: Combining targeted real-world data with advanced analytics to generate evidence at scale in immunology

**DOI:** 10.3389/falgy.2022.951795

**Published:** 2022-11-03

**Authors:** Bernard Hamelin, Paul Rowe, Cliona Molony, Mark Kruger, Robert LoCasale, Asif H. Khan, Juby Jacob-Nara, Dan Jacob

**Affiliations:** ^1^ Sanofi, Paris, France; ^2^ Sanofi, Cambridge, MA, United States; ^3^ Sanofi, Bridgewater, NJ, United States

**Keywords:** electronic health record, machine learning, medical informatics, outcome assessment, Immunolab

## Abstract

Real-world evidence (RWE) has traditionally been used by regulatory or payer authorities to inform disease burden, background risk, or conduct post-launch pharmacovigilance, but in recent years RWE has been increasingly used to inform regulatory decision-making. However, RWE data sources remain fragmented, and datasets are disparate and often collected inconsistently. To this end, we have constructed an RWE-generation platform, Immunolab, to facilitate data-driven insights, hypothesis generation and research in immunological diseases driven by type 2 inflammation. Immunolab leverages a large, anonymized patient cohort from the Optum electronic health record and claims dataset containing over 17 million patient lives. Immunolab is an interactive platform that hosts three analytical modules: the Patient Journey Mapper, to describe the drug treatment patterns over time in patient cohorts; the Switch Modeler, to model treatment switching patterns and identify its drivers; and the Head-to-Head Simulator, to model the comparative effectiveness of treatments based on relevant clinical outcomes. The Immunolab modules utilize various analytic methodologies including machine learning algorithms for result generation which can then be presented in various formats for further analysis and interpretation.

## Introduction

In recent decades, the role of the randomized controlled trial (RCT) in medical decision-making has been elevated to that of “gold standard”. However, from battlefield treatments to the birth of modern epidemiology with John Snow's observational deductions of the role of water pumps during 19th-century cholera outbreaks, medical breakthroughs based on real-world observations long preceded the advent of the modern RCTs. Real-world evidence (RWE) and RCT studies are complementary and reflect evidence from real-life clinical practices compared to controlled environments respectively. RWE is complementary to RCT and can address the disadvantages of both observational studies (e.g., follow-up cost, long study periods, and maintenance of consistency during the study period) and RCTs (e.g., loss of participants at follow up, changes in treatment, long study periods, and cost). Health authorities have historically used these data to generate RWE mainly for post-launch safety, but in recent years, regulatory agencies, payors, and healthcare providers have recognized the benefits of RWE in decision-making for effectiveness as well. Increasingly, RWE is also being considered in other situations such as to construct “pseudo”-control arms (sometimes referred to as “synthetic control arms”) (1–3), and to support regulatory label-expansion applications or effectiveness assessments in situations where RCTs are difficult or unethical to conduct (4, 5). The volume of digitized healthcare data has increased significantly and consequently the potential of RWE to support pharmaceutical development and decision-making is on the rise.

In the United States, the 21^st^ Century Cures Act, passed in December 2016, established public-private partnerships to collect data, improve understanding of diseases, and support patient-focused drug development. It recognized the need for broader, more adaptable decision-making frameworks that incorporate RWE (6, 7). The European Medicines Agency (EMA), the US Food and Drug Administration (FDA), and the Japanese Pharmaceuticals and Medical Devices Agency (PMDA) are all considering greater user of RWE for informing regulatory decision-making, in contexts other than pharmacovigilance and safety, including drug approvals (8–11).

The use of RWE to inform clinical development and medical research has similarly progressed in recent years. Payers analyze transactional claims data to understand clinical populations and impediments to adoption of therapy, and to monitor the effectiveness of providers or standards of care. Pharmaceutical companies use RWE as part of integrated evidence-generation plans, identifying evidence needs across the product life cycle, to advance understanding of treatment patterns, patient subpopulations, comparative effectiveness, and marketplace adoption, and to support value-based reimbursement scenarios. Research and development teams use RWE to supplement clinical findings, and even to inform patient recruitment in RCTs. However, pharmaceutical research has struggled to fully embrace RWE, and disparate datasets, siloed data environments, and the absence of easily accessible analytic tools have slowed the adoption of RWE.

To this end, we have constructed a RWE-generation platform to accelerate evidence generation. This tool aims to demonstrate the value of a “platform strategy”—combining richly curated clinical data, patient cohorts, a variety of analytic techniques, and intuitive dashboards—to help accelerate hypothesis generation using real world data.

## Methods

### Immunolab: an insight-generation platform for the evolving RWE landscape

**Figure 1:**
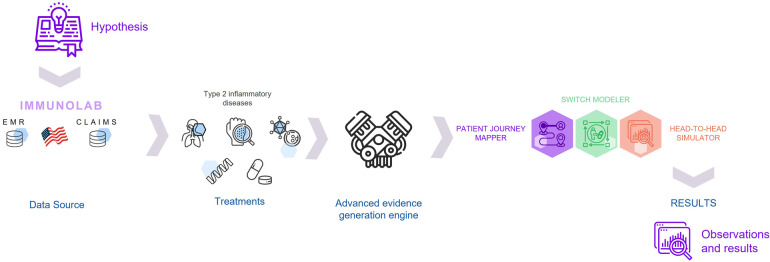
Schematic figure with the different components of the system.

Immunolab is designed to address research questions related to drug development, as well as pre- and post-launch evidence generation needs. This is particularly significant for type 2 inflammatory diseases such as asthma and chronic rhinosinusitis with nasal polyps, which have high prevalence, high rates of comorbidity, and diverse clinical management. Researching type 2 inflammatory diseases requires a “broader” view of data and need of analytic tools to better understand patient treatment journeys, comorbidities, and methods to help predict outcomes. A schematic figure with the different components of the Immunolab platform (hypothesis, data source, treatments, advanced evidence generation engine and results) and how they are connected to each other has been provided in Figure 1.

Immunolab aims to provide the capability to rapidly explore key research questions, with a fundamental consistency built into population cohorts such as demographics, comorbidities, treatments, and outcomes.

### Design approach

A multidisciplinary collaborative team from multiple functions including research, clinical, medical, and health economics and outcomes research was formed, which took part in a workshop and identified key evidence gaps in RWE for immunology research, and based on those gaps research questions were developed which formed the basis of the research framework for Immunolab.. This workshop also confirmed the need for both a “platform solution” and for distributed access to hypothesis-generation tools. The Immunolab core development and analytic design teams thereafter aligned on cohort definitions and feature designs. Scenarios were modeled using RWE to explore the ramifications of key design choices. All design decisions (and scenarios leading to those design decisions) made by the teams were curated in a decision log, which serves as a repository for institutional memory and supports ongoing maintenance of Immunolab.

### Infrastructure

Immunolab was built on a RWE environment, a secure cloud-based system based on a high-performance computing spark cluster, machine learning (ML) libraries, and data visualization tools. The system was designed to be flexible, allowing easy integration of new tools and capabilities as required by the evolving demands of RWE, while maintaining strong data protection and access integrity.

### Optum^®^ de-identified electronic health record (EHR) data source

**Figure 2:**
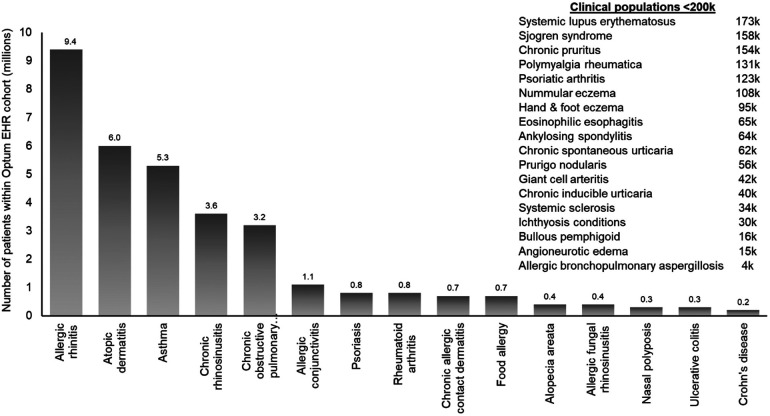
Diseases within the Immunolab real-world data cohort. COPD, chronic obstructive pulmonary disease; EHR, electronic health record; RW, real-world.

The Immunolab platform utilizes patient cohorts, which are regularly updated, from the Optum de-identified EHR dataset (2007–2019). Optum data are de-identified in line with the Health Insurance Portability and Accountability Act statistical de-identification rules and managed according to relevant data use requirements (12, 13). The Optum EHR enables Immunolab to analyze data from over 17 million patients; this large cohort enables detailed investigation of patients with diseases such as asthma, atopic dermatitis (AD), and chronic rhinosinusitis with and without nasal polyps (Figure 2). Within this cohort, 33 type 2 immunological indications and related comorbidities were available.

## Results

### The Immunolab platform interface

Immunolab platform is a user-friendly web-based interface that employs maps, drop-down menus, and intuitive graphs to perform and display analyses. Immunolab allows the user to easily select pre-specified “analytical modules” with a primary purposes of hypothesis generation. Cohorts to be assessed by the respective analytical module can be generated within the Optum EHR dataset by applying automated eligibility criteria based on diagnosis and treatment codes.

### Analytical modules

The three analytic modules of Immunolab were: (i) Patient Journey Mapper (PJM) to describe the drug treatment patterns in patient populations, (ii) Switch Modeler (SM) to model treatment switching pattern and identify its drivers, (iii) and Head-to-Head Simulator (H2H) to model the comparative effectiveness of treatments based on relevant clinical outcomes. For each of these three analytical modules, clinically relevant features were used for both descriptive analyses and modeling, including timing of diagnoses and treatments, demographics, patient characteristics, medical attributes, disease activity, comorbidities, medication, health-care providers, and procedures. The PJM can provide approximately 5 million analyses across the predefined 70 patient subpopulations; the SM can do nearly 130 descriptive statistics for every switch/augmentation event, yielding up to 2 million analyses; and the H2H can do approximately 75,000 descriptive analyses, with up to 150 descriptive statistics for approximately 150 patients subpopulations across four therapeutic groups. Consequently, Immunolab can facilitate over 7 million rapid “insight generation” analyses.

### The Patient Journey Mapper (PJM)

The PJM module descriptively assesses the characteristics and treatment journeys of patients. It provides data driven results for overarching questions of interest such as: “*Which patients are …?*”; “*What is happening to patients who are…?*”; “*What are the treatment journeys …?*”; and “*What are the common combinations of diseases/comorbidities of …?*”.

**Figure 3:**
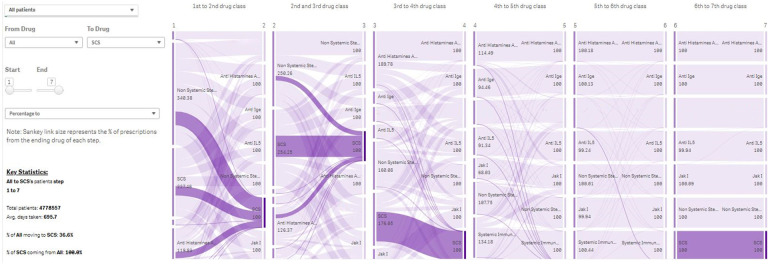
Treatment Journey of all patients in Immunolab from any treatment they are receiving to systemic corticosteroids. **Note:** The PJM provides user-friendly visual output in the form of Sankey plots, describing the use of drug classes across lines of therapy for patient subpopulations of interest. IgE, Immunoglobulin E; IL, interleukin; PJM, Patient Journey Mapper.

The PJM is based on a “patient-quarter” data framework. For example, to identify patients by using the “standard of care” filter in December 2019, the module identifies and selects all patients who have had the standard of care prescription in the 12 months up to the end of December 2019. For subpopulations of interest, “Lift” scores (a measure of distinctiveness, equivalent to the ratio of the prevalence in the subpopulation to the prevalence in the whole population) are calculated. Patient journeys are illustrated by visual displays of the escalation order of the drug classes used in the population of interest, with automated generation of histograms to describe patient characteristics and changes over time; interactive Sankey plots describing the treatment pathways (the collective of treatment switches); and column charts describing disease combinations (Figure 3).

### The Switch Modeler (SM)

There is a limited understanding of the factors that drive switch of patients across drug classes for the treatment of asthma and AD. The SM module is intended to generate evidence on the key factors leading to switching to and from specific treatments based on a large data source. Treatment changes can be categorized as “switching” (discontinuation of a prescription for one drug and the initiation of a prescription intended to treat the same disease within a suitable time window) or “augmentation” (initiation of a prescription for a drug with concurrent continuation of an existing prescription intended to treat the same disease with another drug).

**Figure 4:**
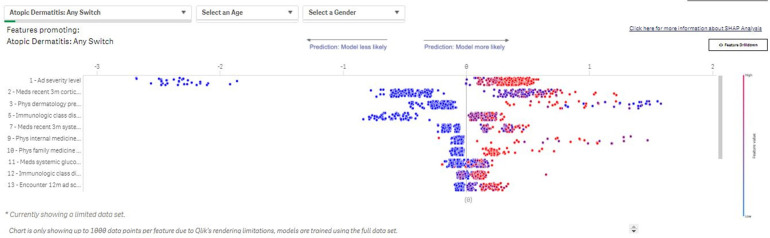
Switch Modeler—Key drivers of patients with AD switching between treatments. The SM uses SHAP to determine drivers of the observed changes in prescribing patterns. The value of each binary variable data point is indicated by color (blue: low/no; red: high/yes). The effects of the covariate on risk prediction are shown in log-odds scale on the horizontal axis; covariates are listed in descending order by relative importance in terms of driving prescribing patterns in the subpopulation of interest. SHAP outputs from each patient included in an analysis are then aggregated to provide population-level SHAP estimates of covariate impact. SHAP, SHapley Additive exPlanation; SM, Switch Modeler.

Based on the SM parameters, an underlying data table is created that contains patient profiles for which the treatment change occurs, as well as patients for which the treatment change does not happen. An ML algorithm, Light Gradient Boosting Machine (LightGBM (14), is trained on this data table and learns to discriminate whether a regimen change will occur for a patient based on the available attributes e.g., demographic profiles, clinical characteristics of disease, and patient clinical phenotype). For patients experiencing a treatment change, the attributes are calculated based on the period leading up to the change. Attributes for patients who do not experience a regimen change are based on the most recent data available. Relevant drivers of the treatment changes are then derived based on the SHapley Additive exPlanation (SHAP) approach, a unified analytical approach to evaluating the output of different ML models (Figure 4). The SHAP value of any single covariate represents its relative effect on the model's prediction. By assessing the full range of covariates in this way, we can see which covariates are, on average, the key drivers of the model's patient-level prediction. Aggregating all model explanations from all patients in a population provides accurate population-level model explanations.

### Head-to-Head simulator (H2H)

To facilitate robust comparisons of different drug classes against each other across clinically relevant endpoints, we need to use analytical adjustment methodologies to correct for confounding effects that may be present in the data. In building the simulator, we considered two approaches: G-estimation (15) and inverse probability of treatment weighting (IPTW) (16, 17). The H2H simulator estimates treatment effects for a series of clinically relevant endpoints. The approaches that were analyzed only consider an endpoint in isolation; no dependency between endpoints was modeled. This means that for a given endpoint (e.g., the number of exacerbations in patients with asthma), either G-estimation or IPTW is used. In either approach, a data table is created that contains a record per treatment exposure. Observed values for the endpoints are calculated over the entire treatment exposure, and covariates are calculated for each treatment exposure based on the period before start of exposure. Both approaches attempt to correct for confounders by including these covariates in the outcome model, for G-estimation, or the propensity model, for IPTW. The outcome model and propensity model are estimated using LightGBM, allowing the models to consider a large set of pre-treatment covariates.

## Future direction

With the increasing availability of digital and digitized healthcare data, and broadening demand for RWE across pharma, standardized methodologies, and platform tools like Immunolab are complementary to the current one-question-at-a-time approach to generating evidence. Unlike RCT methodologies, which have for a long time been under the auspices of practice quality guidelines (such as the International Conference on Harmonisation of Technical Requirements for Registration of Pharmaceuticals for Human Use Good Clinical Practice guidelines) and clinical trial registries (ClinicalTrials.gov and EudraCT.eu), rigorous methods are required to optimize RWE in the regulatory context, and international standardization of using RWE in healthcare decision-making has been attempted only relatively recently (18). In recent years, the US Food and Drug Administration has developed innovative programs (including the Sentinel Initiative) to accelerate the use of RWE to support assessments of safety and to facilitate label extensions. To do so, a full life-cycle RWE platform is needed that would allow exploring these opportunities by generating hypotheses and testing assumptions. Platform such as Immunolab seek to address this issue by using ML protocols to optimize modeling of patient histories, standardizing analytical methodologies, and constructing consistent outputs based on these analyses. Future expansions integrating new analytic modules and additional data sources into platforms such as Immunolab can put the means of rapid analytic exploration into the hands of researchers, making both data and analytics available to them. These platforms can serve as first-line resource for evidence generation, by accelerating the process and addressing important real-world data research questions at a deeper, faster, and more impactful level. They also provide the possibilities to create a cross-academia and cross-industry network of researchers with a focus on improving health outcomes based on big data driven insights.

## Data Availability

The original contributions presented in the study are included in the article/Supplementary Material, further inquiries can be directed to the corresponding author/s.
